# Blue Parotid Unveiled: A Rare Case of Traumatic Hemorrhagic Parotid Lymphangioma in an Eight-Year-Old Boy

**DOI:** 10.7759/cureus.46415

**Published:** 2023-10-03

**Authors:** Muizzuddin Fuad, Bee See Goh, Farah Liana Lokman, Mohd Razif Mohamad Yunus

**Affiliations:** 1 Otolaryngology - Head and Neck Surgery, Universiti Kebangsaan Malaysia Medical Centre, Kuala Lumpur, MYS; 2 Otorhinolaryngology/Pediatrics Otorhinolaryngology, Universiti Kebangsaan Malaysia Medical Centre, Kuala Lumpur, MYS; 3 Otorhinolaryngology - Head and Neck Surgery, Universiti Kebangsaan Malaysia Medical Centre, Kuala Lumpur, MYS

**Keywords:** facial nerve, cavernous lymphangioma, preauricular swelling, neck swelling, parotid lymphangioma

## Abstract

Parotid lymphangioma is a benign lymphatic malformation commonly observed in infancy or early childhood. It often grows insidiously and presents as a painless, soft fluctuant mass. We report a case of an eight-year-old boy who was diagnosed from another center with right parotid lymphangioma of one-year duration. He presented with right painful preauricular swelling and trismus for nine days after a recent history of blunt trauma to the preauricular caused an acute expansion of the swelling and subsequently, the patient developed ipsilateral facial nerve palsy. Examination showed right preauricular swelling measuring about 6 x 6 cm that extended posteriorly until the mastoid region, superiorly until the zygoma, and inferiorly until the angle of the mandible, pushing the ear lobule anteromedially. There was bluish discoloration of the overlying skin. The swelling was warm and tender on palpation. Multiple shotty lymph nodes were palpable at the posterior triangle. Aspiration of the swelling revealed blood content, but it reaccumulated after a few hours. A magnetic resonance imaging (MRI) of the neck showed a lesion confined within the parotid gland. There was a presence of air-fluid level with dependent layers of hyperintense on the T1-weighted image (T1WI) and T2-weighted image (T2WI) with clumps of isointensity on T1WI, which are hypointense on T2WI, which is suggestive of acute-late subacute blood product. A diagnosis of lymphatic malformation complicated with hemorrhage was made. Hence, the patient underwent surgery for the evacuation of blood clot and right superficial parotidectomy. Histopathological examination of the intraoperative tissue biopsy revealed evidence of venolymphatic malformation of the parotid gland. Postoperatively, he was discharged home after three days. The facial nerve function recovered from House and Brackmann grade II to grade I three weeks after the surgery.

## Introduction

Venolymphatic malformations (VLMs) are vascular anomalies that are classified according to their major vessel type, which can be arterial, venous, lymphatic, or mixed [1]. The occurrence of parotid gland vascular malformations is rare, and despite advances in imaging, including magnetic resonance imaging (MRI), the diagnosis is still quite challenging, and the differentials of other parotid gland tumors must be excluded, especially hemangiomas [2]. We present a case of an eight-year-old boy with a painful right parotid swelling complicated with ipsilateral facial nerve palsy that turned out to be VLM evidenced by histopathological examination after the surgical excision of the swelling.

## Case presentation

An eight-year-old boy was diagnosed with right parotid lymphangioma and presented with a painful, acute right preauricular swelling for nine days following blunt trauma to the area. The patient initially received treatment at a private center where intravenous antibiotic was given along with the blind aspiration of the swelling. However, the swelling reaccumulated after three days, hence he was referred to our center. He had similar swelling a year prior, which resolved with aspiration.

**Figure 1:**
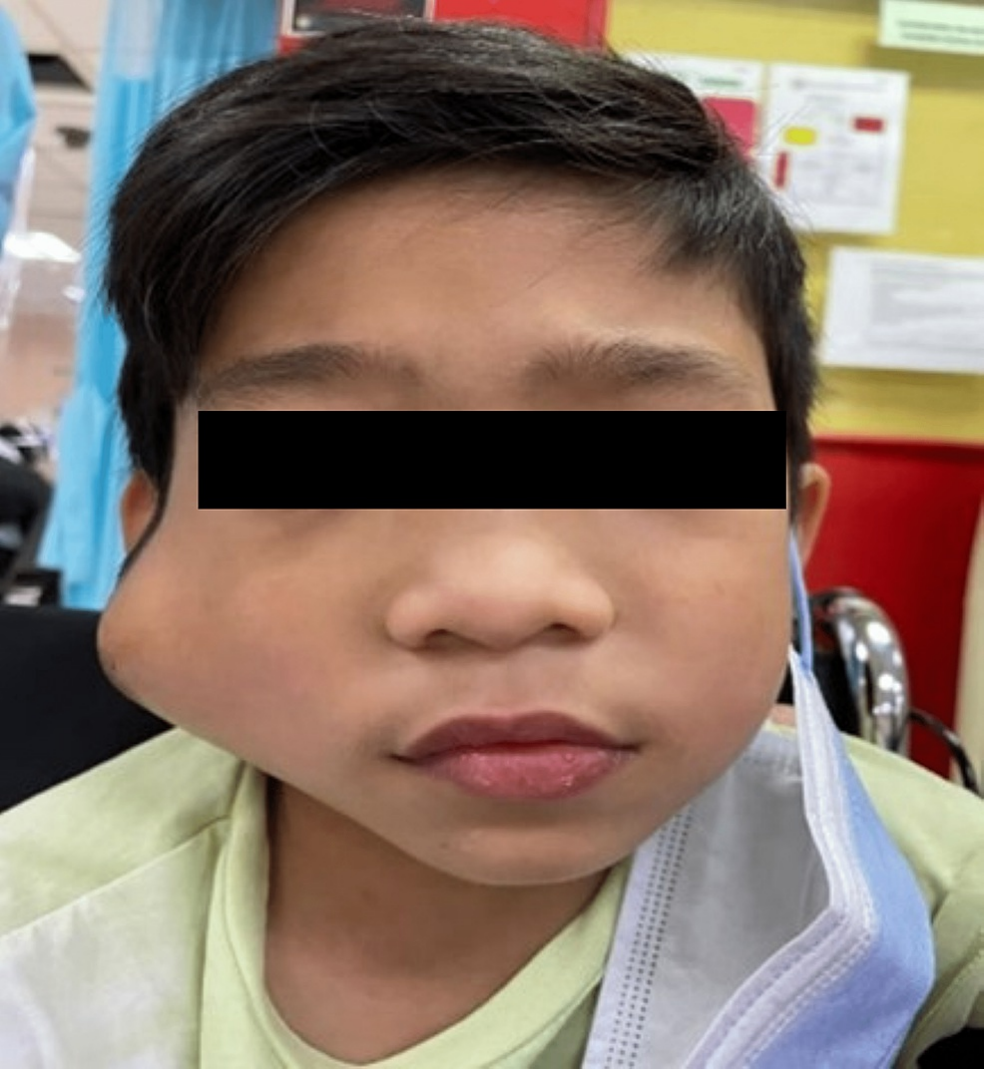
Right parotid swelling measuring 6 x 6 cm.

**Figure 2:**
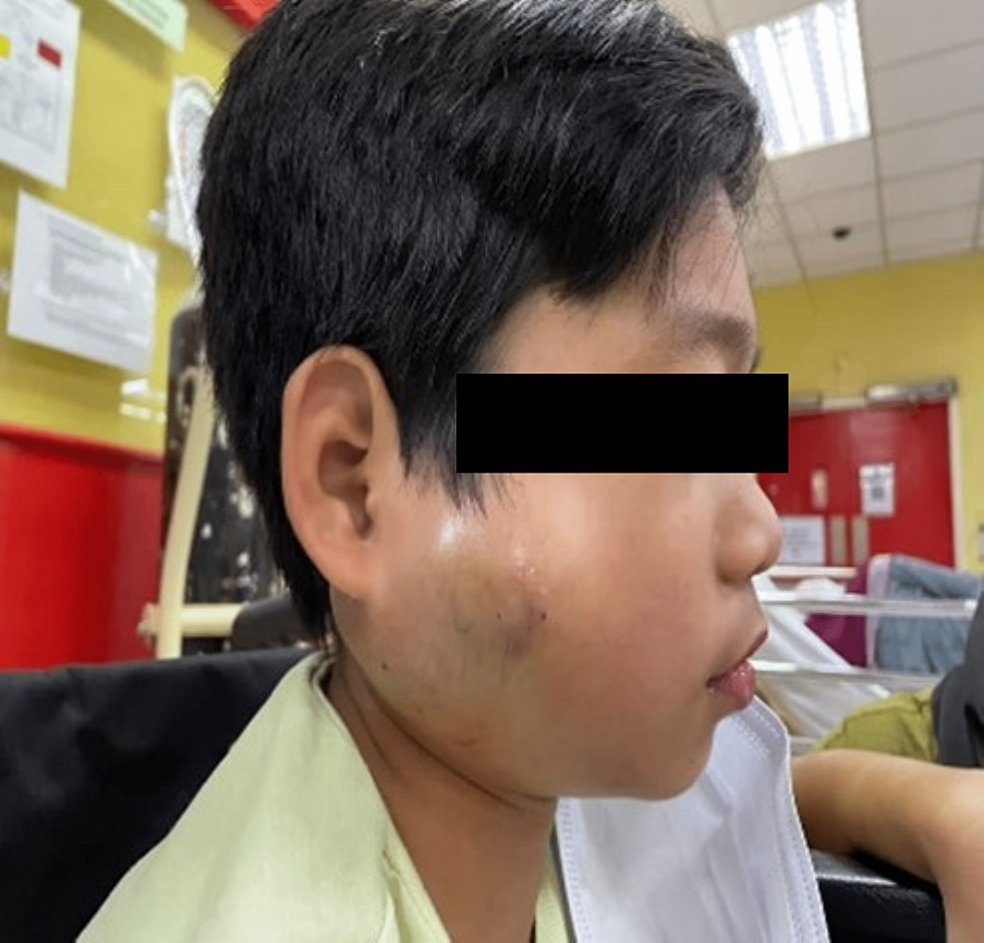
Right parotid swelling that extends anteriorly until mid of the mandible, posteriorly until the mastoid tip, superiorly until the tragus, and inferiorly until the mandibular angle. The overlying skin appears bluish with ecchymosis.

Clinical examination showed swelling at the right preauricular region measuring 6 x 6 cm that extended posteriorly until the mastoid region, superiorly until the zygoma, and inferiorly until the right mandibular angle, pushing the ear lobule anteromedially. There was bluish discoloration of the overlying skin. The swelling was warm and tender on palpation. Multiple shotty lymph nodes were palpable at the posterior triangle (Figures 1, 2). On initial presentation, there was no facial asymmetry and facial nerve functions were normal.

Ultrasound of the right parotid region showed multiloculated multicystic fluid within the swelling. A single vessel was noted within the septations and there was thickening with increased echogenicity of the overlying subcutaneous fat. Color Doppler sonography showed low blood flow within the septa.

**Figure 3:**
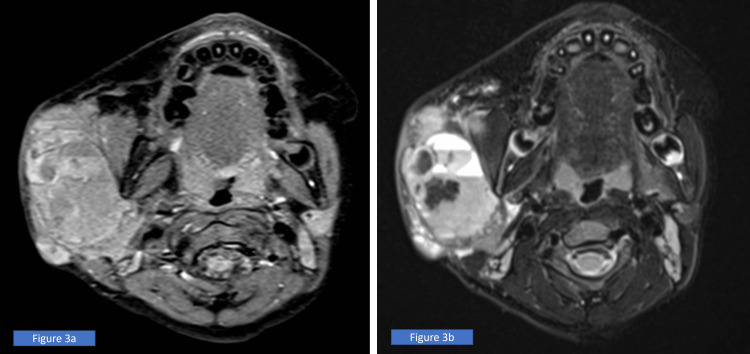
(a) Contrasted MRI T1 and (b) contrasted MRI T2 with an axial view at the level of the angle of the mandible showed a lesion that is confined in the superficial and deep lobe of the parotid with multiple fluid levels and presence of acute and subacute bleeding. There is contrast enhancement predominantly at the inferior portion and septa, with mass effect. No parapharyngeal space, postauricular, or intramuscular extension.

Subsequently, an MRI of the neck was done (Figure 3) to further evaluate the mass and its extension prior to surgical intervention.

**Figure 4:**
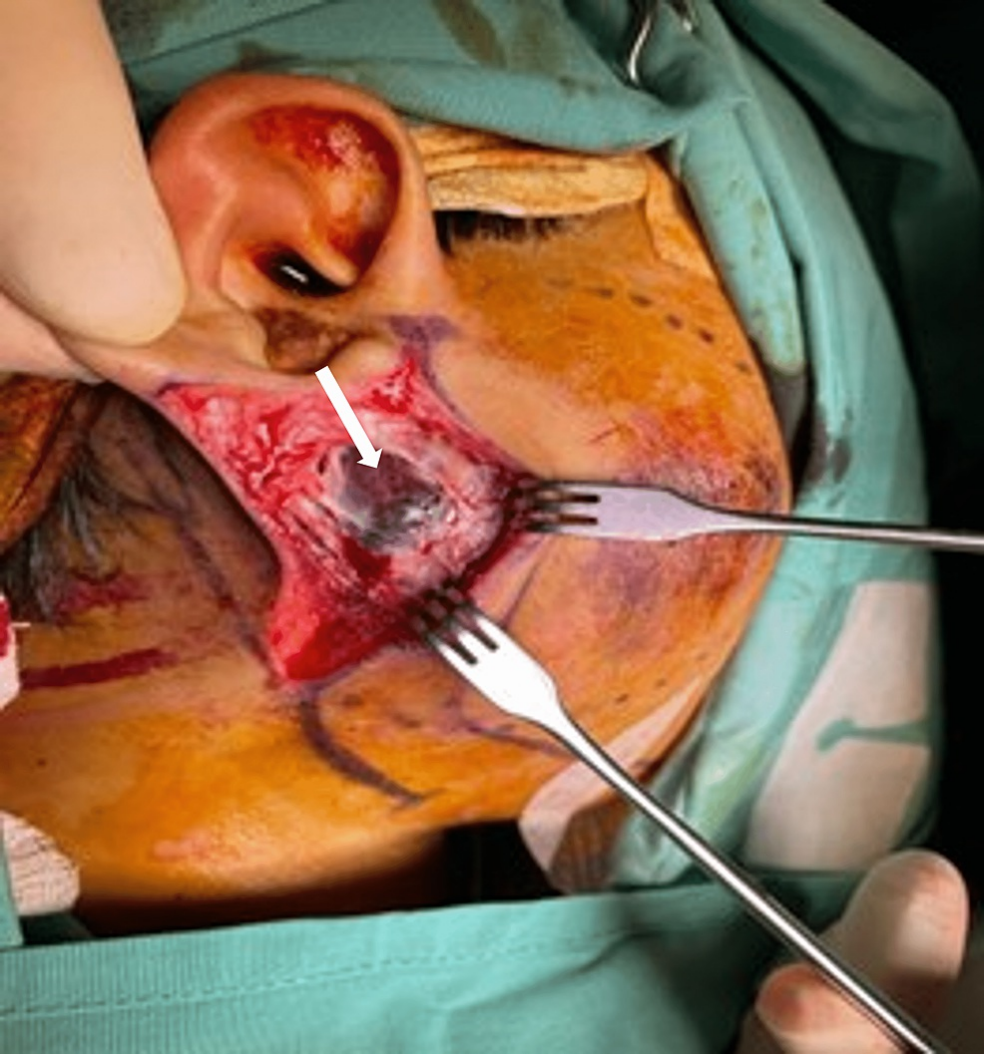
A bluish mass underneath the superficial muscular aponeurotic system (SMAS) in the parotid gland.

**Figure 5:**
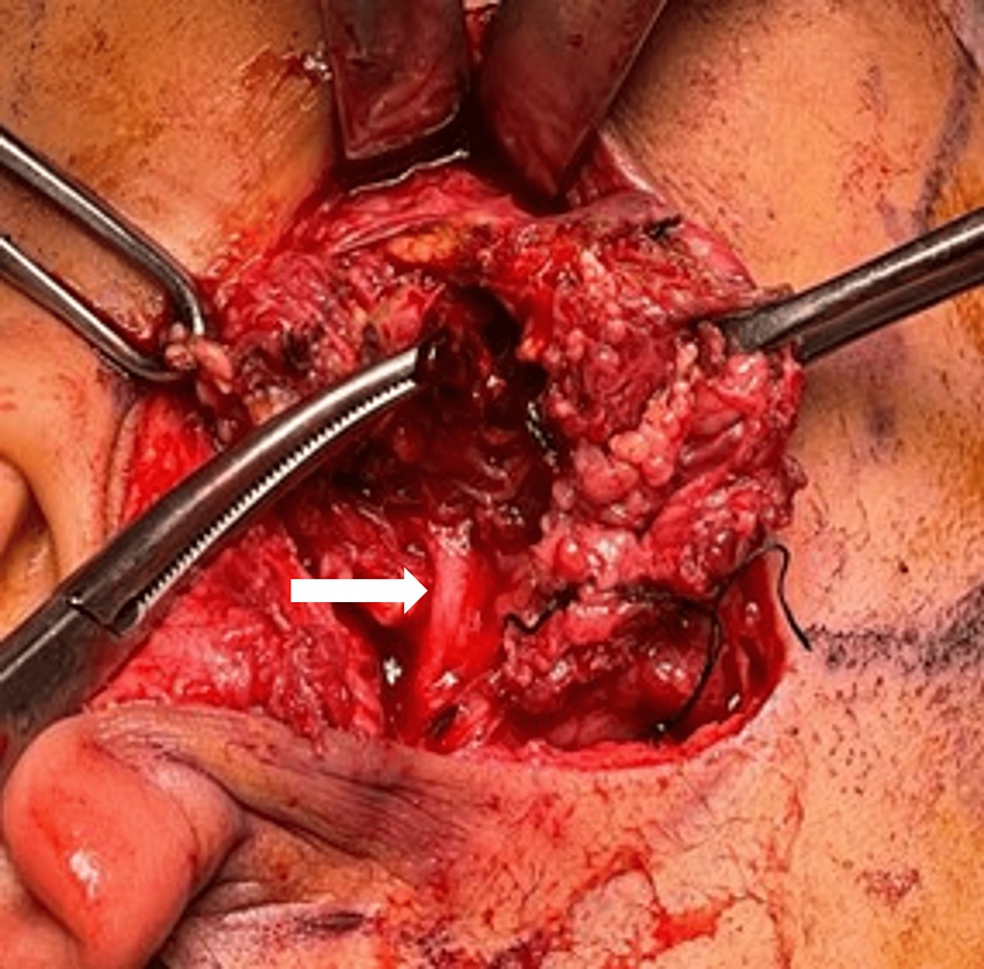
The facial nerve trunk (marked with a white arrow) was identified and preserved.

Ultrasound-guided aspiration was performed with the aim of emergency decompression to reduce the swelling and pain, thus reducing the mass effect. However, the swelling recurred and expanded and after the second aspiration, he developed facial nerve paresis (House and Brackmann grade II). Subsequently, the patient underwent emergency evacuation of the blood clot and right superficial parotidectomy. Intraoperatively, a soft bluish mass was seen upon elevation of the superficial muscular aponeurotic system (SMAS). There were also blood clots within the mass as well as within the superficial parotid gland (Figures 4, 5). Blood clots were evacuated over the superficial parotid gland and proceeded with superficial parotidectomy as the mass was adherent to the parotid gland.

Histopathological examination reported a VLM that displayed benign parotid parenchyma with thin wall lymphatic channels and congested vascular spaces surrounded by fibrous stroma. There was no cytological atypia or evidence of malignancy seen.

Postoperatively, the patient was well and discharged home after three days. He also received facial nerve physiotherapy and eye care. At six weeks postoperatively, facial nerve functions recovered fully.

## Discussion

VLMs are rare, benign, congenital vascular anomalies that present at birth but are usually asymptomatic and manifest in late childhood or early adulthood [3]. It is a mixed vascular malformation, according to the International Society for the Study of Vascular Anomalies (ISSVA), comprising both veins and lymphatic vessels [4]. Venous malformations (VMs) account for half of all vascular malformations. Lymphatic malformations (LMs) are rare, as are combined LMs and VMs [5]. VLM has a 1% prevalence and an estimated incidence of one to two per 10,000 births [6].

These vascular malformations are most commonly found in the head and neck (60%), but the incidence of parotid gland VLM is very rare [1,7]. Capillary, venous, and lymphatic malformations are slow-flow malformations, whereas arteriovenous malformations (AVMs) are fast-flow malformations [8].

VMs are mostly asymptomatic, but patients may experience symptoms such as bluish discoloration of the skin, pain, swelling, and compression of neighboring structures, such as the facial nerve, which results in facial nerve paralysis when it grows to a significant size, as well as esthetic deformities [6].

Ultrasonography is commonly utilized as the first imaging modality for suspected VM cases because of its low cost, broad availability, and absence of ionizing radiation, which is especially important considering the high prevalence of lesions in the pediatric population [9]. The use of ultrasonography is critical in distinguishing between high-flow AVMs and low-flow lesions such as venous or lymphatic malformations [10].

The distinction between venous and lymphatic malformations in ultrasound is that the LM typically exhibits hypoechoic, thin-walled, multiloculated, and multicystic swelling, and color Doppler ultrasonography may discern between the two, while in the latter, usually no blood flow can be observed [9,11]. The presence of phleboliths and veins crossing the lesion can be detected in VM. However, ultrasound alone is not sufficient prior to surgical intervention as it is unable to determine the anatomical planes for resection or deeper extension of the lesion.

A CT scan can also demonstrate the involvement of the parotid gland's deep lobe and an enhancing pattern of the lesion. For best VM visualization, MRI is the diagnostic modality of choice [12]. The presence of septations and tortuous slow-flow veins, as well as phleboliths with a hyperintense T2-weighted signal on MRI, is characteristic of VM [1].

Histopathological examination is needed to confirm the diagnosis, revealing dilated ectatic venules bordered by flat endothelium lining and lymphatic arteries with patchy smooth muscle in the wall. Vessels may be congested, thrombosed, or calcified (phleboliths). Proteinaceous particles may be found in lymphatic channels [1].

The treatment of VMs is determined by their size, location, symptoms, and closeness to critical structures. Furthermore, because VMs have poorly defined boundaries and a proclivity to invade normal tissues, they necessitate cautious treatment decisions to protect the surrounding architecture [1]. A multidisciplinary approach is the standard of care in the treatment of VM [13]. Management can be challenging due to the high possibility of functionally essential structures being involved. Medical treatment, sclerotherapy, embolization techniques, and surgery are among the options for treating VMs of the head and neck [14].

Aspiration of the swelling can be done as an emergency decompression in case of very painful swelling or rapid enlargement causing compression of vital structures. However, aspiration of suspected vascular tumors must be done cautiously due to the risk of bleeding. It is always preferable for aspiration to be done under ultrasound-guided rather than a blunt aspiration as the risk for complications is higher, especially in the pediatric age group. Bhatt et al. (2018) reported a case of parotid swelling that underwent ultrasound-guided aspiration for decompression that was subsequently complicated with hemorrhage causing pain and facial nerve palsy in a 24-year-old gentleman with parotid LM [15]. We reported a case of parotid swelling in an eight-year-old boy who underwent blunt aspiration at a private clinic, which predisposed him to a significant risk of bleeding and additional complications.

According to Achache et al. (2013), preoperative needle aspiration and imaging are unable to conclude the final diagnosis owing to the rare occurrence of tumor or vascular malformations in the parotid gland. Hence, the surgical excision of the parotid gland with preservation of the facial nerve is mainly used to treat patients with suspected VMs of the parotid gland [2]. Surgery can offer both final diagnosis and treatment for the patient. Complete surgical excision is crucial to avoid recurrence. Best surgical outcomes can be obtained by optimal hemostasis, thorough dissection, and wide field exposure to identify critical structures such as facial nerve that is at risk for injury during surgical resection of the tumor [16].

VM can potentially become advanced or aggressive, necessitating facial nerve dissection. Surgery in this scenario may be more challenging, necessitating surgical expertise and skills [2]. Possible complications post-surgical excision include bleeding, fluid collection at the resection site, lymphatic discharge, wound infection, parotid fistula, and recurrence. Hence, postoperatively, the patient should be monitored for complications and subjected to facial nerve physiotherapy for postoperative functional recovery [17]. Repeated MRI post surgery should be scheduled to monitor for recurrence of the VM.

Sclerotherapy is another option for the treatment of VM. Sclerosants such as ethanol, sodium tetradecyl sulfate, and bleomycin are employed and can be administered intraoperatively or under imaging guidance [18]. It works by damaging the lesions' vascular endothelial cells while maximizing the sclerosant concentration and duration within the vessel lumen. It works well for low-flow VMs [18]. However, because of morbidity associated with the sclerotherapy treatment, this therapeutic strategy should be reserved for use by professionals with competence in facilities with experience in the care and management of VM [19]. Further long-term evaluation, particularly for chronic morbidity and sequelae from sclerotherapy, is very necessary.

## Conclusions

In cases of suspected vascular malformation, aspiration of the swelling should be done cautiously under ultrasound guidance, particularly in the pediatric age group. Blunt aspiration may carry a risk of bleeding, expansion of the swelling with compression of vital structures, such as facial nerve, and also causing pain to the patient. The diagnosis of parotid gland VM can be challenging because of the rare occurrence. A high level of suspicion is required, especially in individuals who present with swelling and blue skin discoloration. Hence, imaging and postoperative histopathology results are crucial to make the final diagnosis. Management of VM must be tailored according to the patients. Surgery remains the gold standard for the treatment of vascular malformations of the parotid gland, as it can offer both histopathologic diagnosis and treatment.
